# Two new species and one new record of *Ischnothyreus* Simon, 1893 (Araneae, Oonopidae) from China

**DOI:** 10.3897/BDJ.12.e122100

**Published:** 2024-04-11

**Authors:** Chenxue Song, Yanfeng Tong, Dongju Bian, Zhisheng Zhang

**Affiliations:** 1 Life Science College, Shenyang Normal University, Shenyang 110034, China Life Science College, Shenyang Normal University Shenyang 110034 China; 2 Key Laboratory of Forest Ecology and Management, Institute of Applied Ecology, Chinese Academy of Sciences, Shenyang 110016, China Key Laboratory of Forest Ecology and Management, Institute of Applied Ecology, Chinese Academy of Sciences Shenyang 110016 China; 3 Key Laboratory of Eco-environments in Three Gorges Reservoir Region (Ministry of Education), School of Life Sciences, Southwest University, Chongqing 400715, China Key Laboratory of Eco-environments in Three Gorges Reservoir Region (Ministry of Education), School of Life Sciences, Southwest University Chongqing 400715 China

**Keywords:** Asia, goblin spiders, new distribution, taxonomy

## Abstract

**Background:**

*Ischnothyreus* Simon, 1893 is a large genus of oonopid spiders that currently contains 126 species, amongst which, 28 have been recorded in China.

**New information:**

Two new *Ischnothyreus* species, *Ischnothyreusdaheling* Tong & Zhang, **sp. nov**. and *Ischnothyreuslongyang* Tong & Zhang, **sp. nov**., are described, based on specimens collected from Yunnan Province and *Ischnothyreusvelox* Jackson, 1908 is recorded in China for the first time, based on material collected from Guangxi Province. All three species are illustrated.

## Introduction

Oonopidae is a diverse spider family with 1940 extant described species in 115 genera (WSC 2024). They have a nearly worldwide distribution, occurring mainly in the leaf litter, under bark and in the tree canopy (Jocqué and Dippenaar-Schoeman 2006, Ubick and Dupérré 2017).

The genus *Ischnothyreus* Simon, 1893 is one of the most speciose genera of Oonopidae, with 126 extant species mainly distributed in the Old World (Fu et al. 2023, Tong et al. 2023, WSC 2024). The genus *Ischnothyreus* Simon, 1893 can be recognised by the presence of leg spines, the usually small abdominal scutum, the strongly sclerotised male palps, the heavily sclerotised male endites and the winding genital tube in females (Kranz-Baltensperger 2011).

In this paper, two new species of *Ischnothyreus*, *Ischnothyreusdaheling* **sp. nov**. and *Ischnothyreuslongyang* **sp. nov**. are described from Yunnan, China. Furthermore, *Ischnothyreusvelox* Jackson, 1908 is newly recorded from China (Guangxi).

## Materials and methods

The specimens were examined using a Leica M205C stereomicroscope. Details were studied using an Olympus BX51 compound microscope. Photos were made with a Canon EOS 750D zoom digital camera (18 megapixels) mounted on an Olympus BX51 compound microscope. Endogynes were cleared in lactic acid. Scanning electron microscope (SEM) images were taken under high vacuum with a Hitachi S-4800 after critical point drying and gold-palladium coating. All measurements were taken using an Olympus BX51 compound microscope and are in millimetres. Material is deposited in Shenyang Normal University (SYNU) in Liaoning, China.

The following abbreviations are used in the text and figures: ALE = anterior lateral eyes; bss = bell-shaped structure; fp = flag-shaped process; PLE = posterior lateral eyes; PME = posterior median eyes; rl = retrolateral lobe; stp = strong, tooth-like projection.

## Taxon treatments

### Ischnothyreus daheling

Tong & Zhang sp. nov.

3C8C3265-4398-51D8-BDD7-6EA5214F57E6

0457642A-703E-4A20-AC1B-FFF54BD7A6F1

#### Materials

- **Type status:** Holotype. **Occurrence:** recordNumber: SYNU-707; recordedBy: Zongxu Li, Luyu Wang; individualCount: 1; sex: male; lifeStage: adult; occurrenceID: A9E7AAC7-43DA-5861-B070-AA367E644B62; **Taxon:** scientificName: *Ischnothyreusdaheling*; order: Araneae; family: Oonopidae; genus: Ischnothyreus; **Location:** country: China; stateProvince: Yunnan; county: Baoshan City, Tengchong County; locality: JietouTown, Datou Village, Dahelingganjiao; **Identification:** identifiedBy: Yanfeng Tong; **Event:** samplingProtocol: sifting leaf litter; eventDate: 23/02/2011
- **Type status:** Paratype. **Occurrence:** recordNumber: SYNU-708-710; recordedBy: Zongxu Li, Luyu Wang; individualCount: 3; sex: female; lifeStage: adult; occurrenceID: 0FD72221-D529-5328-9438-36EDCECCEE10; **Taxon:** scientificName: *Ischnothyreusdaheling*; order: Araneae; family: Oonopidae; genus: Ischnothyreus; **Location:** country: China; stateProvince: Yunnan; county: Baoshan City, Tengchong County; locality: JietouTown, Datou Village, Dahelingganjiao; **Identification:** identifiedBy: Yanfeng Tong; **Event:** samplingProtocol: sifting leaf litter; eventDate: 23/02/2011
- **Type status:** Paratype. **Occurrence:** recordNumber: SYNU-711-714; recordedBy: Zongxu Li, Luyu Wang; individualCount: 4; sex: female; lifeStage: adult; occurrenceID: A39B8642-53DA-5493-82BD-1968E6544502; **Taxon:** scientificName: *Ischnothyreusdaheling*; order: Araneae; family: Oonopidae; genus: Ischnothyreus; **Location:** country: China; stateProvince: Yunnan; county: Baoshan City, Tengchong County; locality: Jietou Town, Datang Village; **Identification:** identifiedBy: Yanfeng Tong; **Event:** samplingProtocol: sifting leaf litter; eventDate: 21/02/2011
- **Type status:** Paratype. **Occurrence:** recordNumber: SYNU-715-717; recordedBy: Zongxu Li, Luyu Wang; individualCount: 3; sex: female; lifeStage: adult; occurrenceID: F489D23E-11EB-5FC1-ACD9-A3F0EBA8AB8B; **Taxon:** scientificName: *Ischnothyreusdaheling*; order: Araneae; family: Oonopidae; genus: Ischnothyreus; **Location:** country: China; stateProvince: Yunnan; county: Baoshan City, Tengchong County; locality: Wuhe Town, Xiaodifang Village; **Identification:** identifiedBy: Yanfeng Tong; **Event:** samplingProtocol: sifting leaf litter; eventDate: 27/02/2011
- **Type status:** Paratype. **Occurrence:** recordNumber: SYNU-718; recordedBy: Zongxu Li, Luyu Wang; individualCount: 1; sex: female; lifeStage: adult; occurrenceID: 6DFAC351-F3D7-5037-8495-2316D4E4E770; **Taxon:** scientificName: *Ischnothyreusdaheling*; order: Araneae; family: Oonopidae; genus: Ischnothyreus; **Location:** country: China; stateProvince: Yunnan; county: Baoshan City, Tengchong County; locality: Jietou Town, Sabadi; **Identification:** identifiedBy: Yanfeng Tong; **Event:** samplingProtocol: sifting leaf litter; eventDate: 25/05/2011
- **Type status:** Paratype. **Occurrence:** recordNumber: SYNU-719; recordedBy: Ping Feng; individualCount: 1; sex: female; lifeStage: adult; occurrenceID: AC048212-9E5A-59B5-8D18-EFB379F11EDE; **Taxon:** scientificName: *Ischnothyreusdaheling*; order: Araneae; family: Oonopidae; genus: Ischnothyreus; **Location:** country: China; stateProvince: Yunnan; locality: Gaoligong Mountain, Nankang Protection Station; **Identification:** identifiedBy: Yanfeng Tong; **Event:** eventDate: 20/04/2011

#### Description

**Figure 1.**
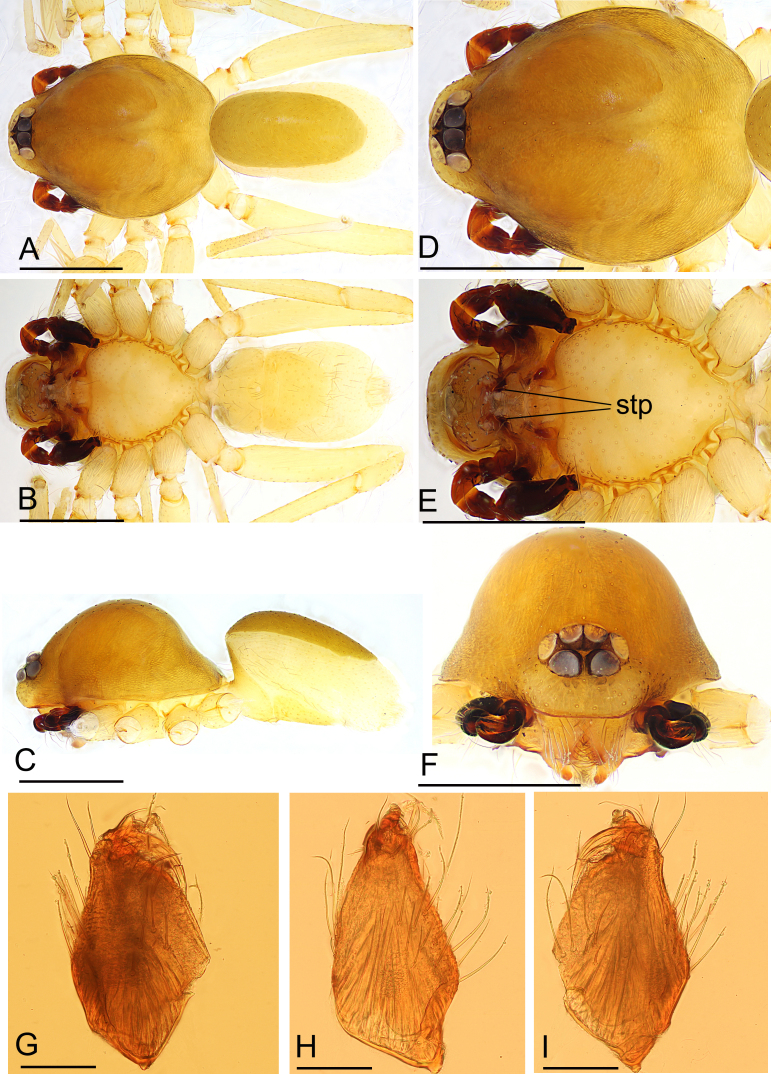
*Ischnothyreusdaheling* sp. nov., male holotype. **A** habitus, dorsal view; **B** habitus, ventral view; **C** habitus, lateral view; **D** prosoma, dorsal view; **E** prosoma, ventral view; **F** prosoma, anterior view; **G** left chelicera, anterior view; **H** left chelicera, lateral view; **I** left chelicera, posterior view. Abbreviation: stp = strong, tooth-like projection. Scale bars: A–F = 0.4 mm; G–I = 0.1 mm.

**Figure 2.**
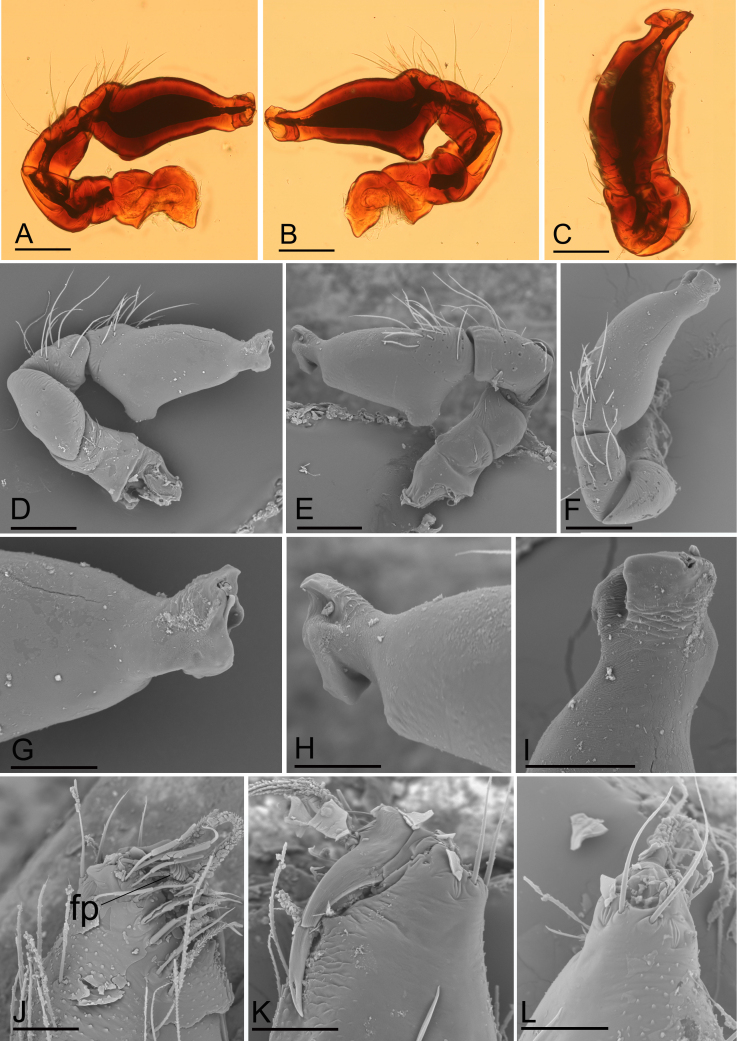
*Ischnothyreusdaheling* sp. nov., male holotype, light (A–C) and SEM (D–L) images. **A** left palp, prolateral view; **B** left palp, retrolateral view; **C** left palp, dorsal view; **D** left palp, prolateral view; **E** left palp, retrolateral view; **F** left palp, dorsal view; **G** distal part of palpal bulb, prolateral view; **H** distal part of palpal bulb, retrolateral view; **I** distal part of palpal bulb, dorsal view; **J** left chelicera, anterior view; **K** left chelicera, posterior view; **L** left chelicera, lateral view. Abbreviation: fp = flag-shaped process. Scale bars: A–F = 0.4 mm; G–L = 0.1 mm.

**Male (Holotype). Body**: habitus as in Fig. 1A–C; body length 1.64. **Carapace**: 0.88 long, 0.65 wide; yellow, oval in dorsal view, pars cephalica strongly elevated in lateral view, sur­face of elevated portion of pars cephalica reticulate, sides finely reticulate, lateral margin straight, smooth (Fig. 1C). **Clypeus**: straight in frontal view, ALE separated from edge of carapace by 1.5 times of their diameter (Fig. 1F). **Eyes**: ALE largest, ALE circular, PME circular, PLE oval; posterior eye row recurved from above; ALE separated by less than their radius, ALE and PLE touching. **Sternum**: as long as wide, pale orange (Fig. 1E). **Mouthparts**: chelicerae, endites and labium yellow; chelicerae straight, base of fangs with flag-like process, fang groove with a few small and one larger denticles (Fig. 2J–L); an­teromedian tip of endites with one strong, tooth-like projection (Fig. 1G). **Abdomen**: 0.76 long, 0.44 wide; dorsal scutum well sclerotised, dark brown, covering 4/5 of the abdomen width and approximately 5/6 of the abdomen length, not fused to epigastric scutum; epigastric and postepigastric scutum pale orange, fused, covering 3/4 of the abdomen length. **Legs**: pale orange, femur I with 2 prolateral spines, tibia I with 4 pairs, metatarsus I with 2 pairs of long ventral spines. Leg II spination similar to leg I, except femur with only 1 prolateral spine. Legs III and IV spineless. **Palp**: trochanter without ventral projection; bulb with 1 ven­tral protuberance, distal end of bulb stout (Fig. 2A–I).

**Figure 3.**
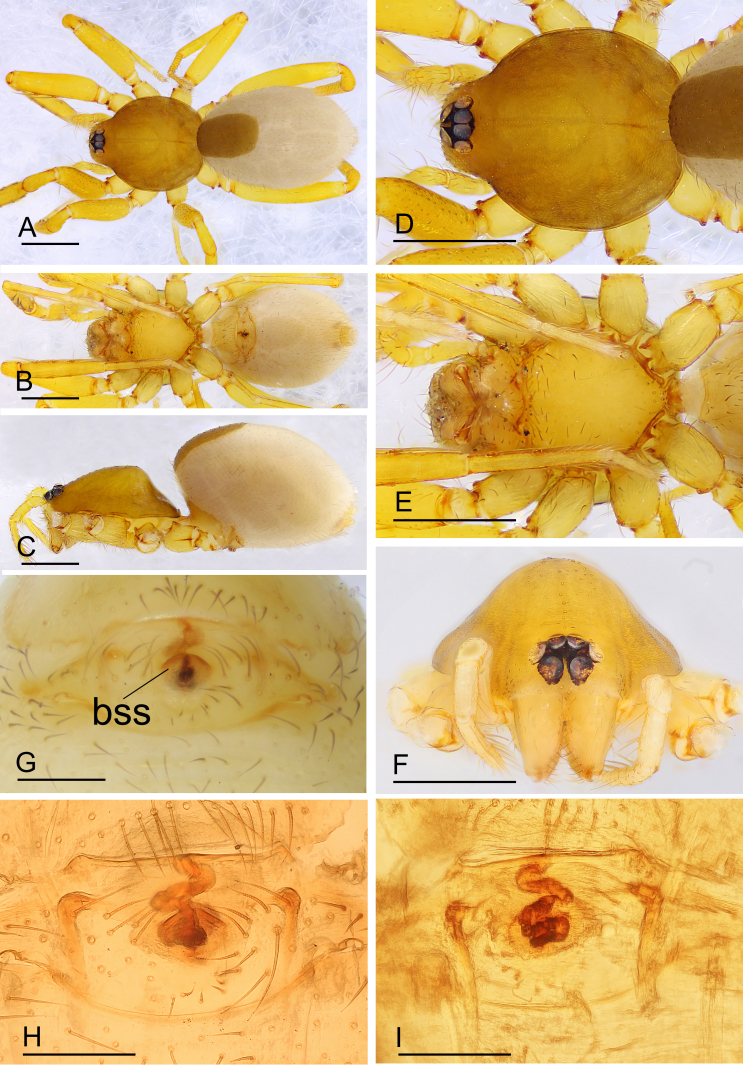
*Ischnothyreusdaheling* sp. nov., female paratype. **A** habitus, dorsal view; **B** habitus, ventral view; **C** habitus, lateral view; **D** prosoma, dorsal view; **E** prosoma, ventral view; **F** prosoma, anterior view; **G** epigastric region, ventral view **H** endogyne, ventral view; **I** endogyne, dorsal view. Abbreviation: bss = bell-shaped structure. Scale bars: A–F = 0.4 mm; G–I = 0.1 mm.

**Female (paratype, SYNU-708).** Same as male, except as noted. Habitus as in Fig. 3A–C. **Body**: 1.82 long. **Carapace**: 0.81 long, 0.66 wide. **Mouthparts**: chelicerae and endites unmodified. **Abdomen**: 1.24 long, 0.79 wide; dorsal scutum approximately 1/3 of the abdomen length. **Epigastric area**: postepigastric scutum with inverted bell-shaped structure (Fig. 3G and H). **Endogyne**: winding tube simple, strongly convoluted; lateral apodemes present (Fig. 3I).

#### Diagnosis

This species is similar to *Ischnothyreusyueluensis* Yin & Wang, 1984 in the size of the abdominal scuta, but can be distinguished by the flag-shaped sclerotised process of cheliceral fang (Fig. 2J) vs. small stick-shaped sclerotised process (Huang et al. 2021: fig. 4G), the stout distal end of bulb (Fig. 2G and H) vs. elongated distal end, with several leaf-shaped membranes (Huang et al. 2021: figs. 5G and H) and the inverted bell-shaped structure of postepigastric scutum of female (Fig. 3G) vs. small, narrow bowl-shaped structure (Huang et al. 2021: fig. 6H).

#### Etymology

The specific epithet is a noun in apposition taken from the type locality.

#### Distribution

Known only from the type locality.

### Ischnothyreus longyang

Tong & Zhang sp. nov.

6632E758-B96B-589E-926F-D3B440D3034D

E2BDA5A7-657E-4266-AF1B-F5A5EC00B662

#### Materials

- **Type status:** Holotype. **Occurrence:** recordNumber: SYNU-721; recordedBy: Zongxu Li, Luyu Wang; individualCount: 1; sex: male; lifeStage: adult; occurrenceID: 57B4E8C2-B0A4-5835-8ED2-92C6C567A3F6; **Taxon:** scientificName: *Ischnothyreuslongyang*; order: Araneae; family: Oonopidae; genus: Ischnothyreus; **Location:** country: China; stateProvince: Yunnan; county: Baoshan City; locality: Longyang District, Lujiang Town, Nankang bealock,; **Identification:** identifiedBy: Yanfeng Tong; **Event:** samplingProtocol: sifting leaf litter; eventDate: 28/02/2011

#### Description

**Figure 4.**
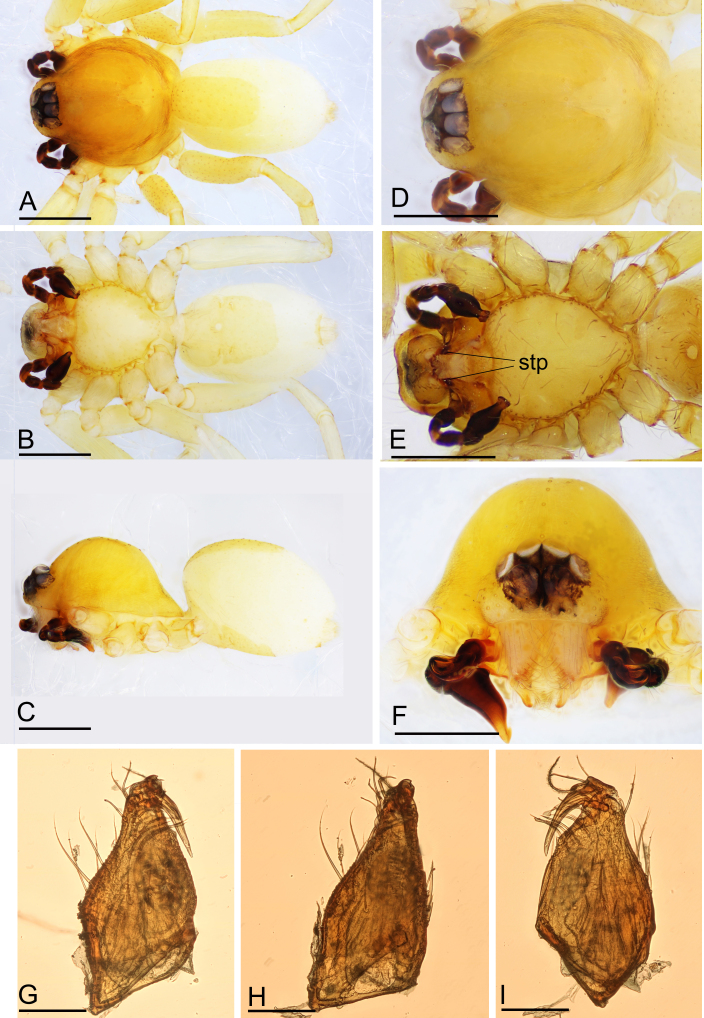
*Ischnothyreuslongyang* sp. nov., male holotype. **A** habitus, dorsal view; **B** habitus, ventral view; **C** habitus, lateral view; **D** prosoma, dorsal view; **E** prosoma, ventral view; **F** prosoma, anterior view; **G** left chelicera, anterior view; **H** left chelicera, lateral view; **I** left chelicera, posterior view. Abbreviation: stp = strong, tooth-like projection. Scale bars: A–F = 0.4 mm; G–I = 0.1 mm.

**Figure 5.**
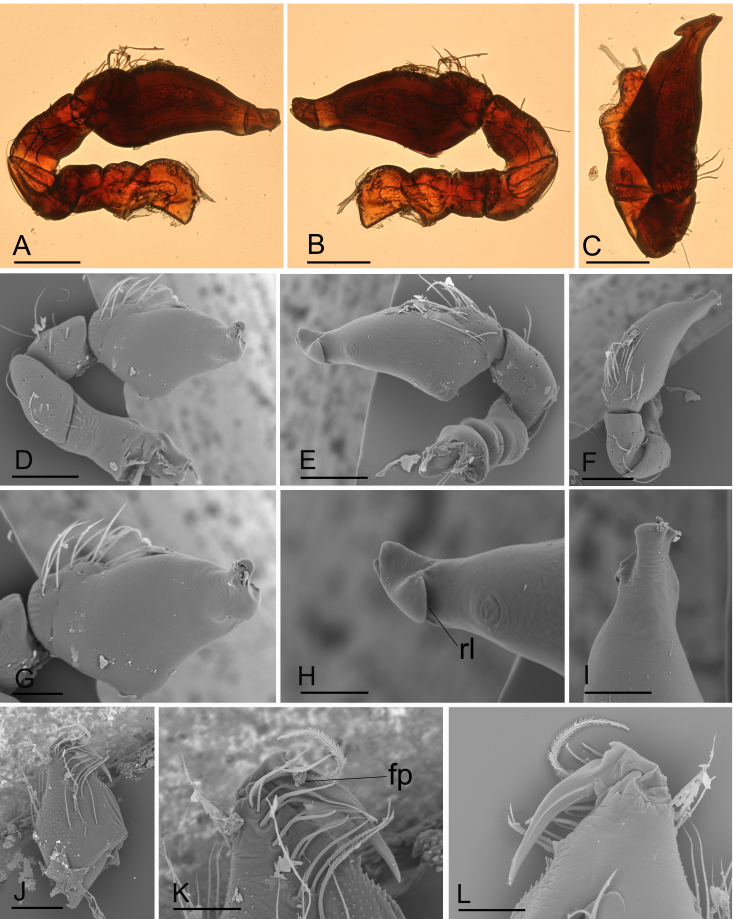
*Ischnothyreuslongyang* sp. nov., male holotype, light (A–C) and SEM (D–L) images. **A** left palp, prolateral view; **B** left palp, retrolateral view; **C** left palp, dorsal view; **D** left palp, prolateral view; **E** left palp, retrolateral view; **F** left palp, dorsal view; **G** palpal bulb, prolateral view; **H** distal part of palpal bulb, retrolateral view; **I** distal part of palpal bulb, dorsal view; **J** left chelicera, anterior view; **K** distal part of left chelicera, anterior view; **L** distal part of left chelicera, posterior view. Abbreviation: fp = flag-shaped process; rl = retrolateral lobe. Scale bars: A–F, J–L = 0.1 mm; G–I = 0.05 mm.

**Male (holotype). Body**: habitus as in Fig. 4A–C; body length 1.91. **Carapace**: 0.91 long, 0.73 wide; yellow, oval in dorsal view, with brown egg-shaped patches behind eyes, pars cephalica strongly elevated in lateral view, sur­face of elevated portion of pars cephalica smooth, sides finely reticulate, lateral margin straight, smooth (Fig. 4C and D). **Clypeus**: straight in frontal view, ALE separated from edge of carapace less than their diameter (Fig. 4F). **Eyes**: ALE largest, ALE circular, PME circular, PLE oval; posterior eye row straight from above; ALE touching, ALE-PLE touching. **Sternum**: longer than wide, pale orange (Fig. 4E). **Mouthparts**: chelicerae, endites and labium yellow; chelicerae straight, base of fangs with flag-like process, fang groove with a few small and two larger denticles (Fig. 5K and L); an­teromedian tip of endites with one strong, tooth-like projection (Fig. 4E). **Abdomen**: 1.00 long, 0.61 wide; dorsal scutum well sclerotised, dark brown, covering 1/2 of the abdomen width and approximately 1/2 of the abdomen length, not fused to epigastric scutum; epigastric and postepigastric scutum well sclerotised, yellow, fused; postepigastric scutum covering about 2/3 of the abdomen length. **Legs**: pale orange, femur I with 2 prolateral spines, tibia I with 4 pairs, metatarsus I with 2 pairs of long ventral spines. Leg II spination similar to leg I, except femur with only 1 prolateral spine. Legs III and IV spineless. **Palp**: trochanter without ventral projection; bulb with 1 ven­tral protuberance, distal end of bulb stout, with a retrolateral broad lobe (Fig. 5A–I).

**Female.** Unknown.

#### Diagnosis

The new species is similar to *Ischnothyreusqiuxing* Tong & Li, 2021 in the size of the abdominal scuta, but can be distinguished by the flag-shaped sclerotised process of cheliceral fang (Fig. 5K) vs. finger-shaped process (Tong et al. 2021: figs. 13H–J, 21F and G) and the broad retrolateral lobe of palpal bulb (Fig. 5H) vs. lack the lobe, but has a tuber-like projection (Tong et al. 2021: fig. 14H).

#### Etymology

The specific epithet is a noun in apposition taken from the type locality.

#### Distribution

Known only from the type locality.

### Ischnothyreus velox

Jackson, 1908

6BF976D3-58AE-5A24-94C6-308F77F4A012

#### Materials

- **Type status:** Other material. **Occurrence:** recordNumber: SYNU-700; recordedBy: Zhigang Chen & Zhe Zhao; individualCount: 1; sex: female; lifeStage: adult; occurrenceID: AE872332-BE8E-5D62-900A-5AD15212C5FB; **Taxon:** scientificName: *Ischnothyreusvelox* Jackson, 1908; order: Araneae; family: Oonopidae; genus: Ischnothyreus; **Location:** country: China; stateProvince: Guangxi; county: Liuzhou City; locality: Hills behind Bus Master Station, Laohuchong, Bajiao cave; verbatimElevation: 85 m; verbatimCoordinates: 24°17'49.5''N, 109°24'10.3''E; **Identification:** identifiedBy: Yanfeng Tong; **Event:** eventDate: 10/12/2012

#### Description

See Platnick et al. (2012).

#### Diagnosis

**Figure 6.**
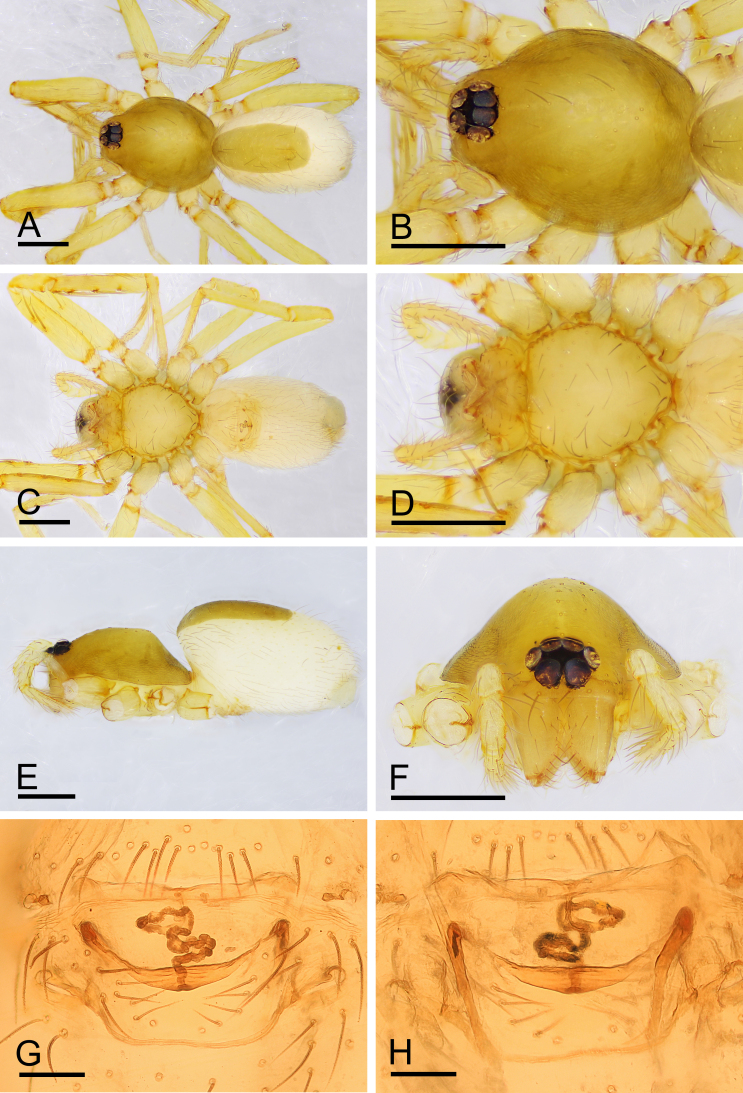
*Ischnothyreusvelox* Jackson, 1908, female. **A** habitus, dorsal view; **B** prosoma, dorsal view; **C** habitus, ventral view; **D** prosoma, ventral view; **E** habitus, lateral view; **F** prosoma, anterior view; **G** endogyne, ventral view; **H** endogyne, dorsal view. Scale bars: A–F = 0.4 mm; G, H = 0.1 mm.

Males differ from those of *Ischnothyreuspeltifer* (Simon, 1892) in lacking a protuberance on the base of the fang; palp with rounded bulb and sperm pore larger (Platnick et al. 2012: figs. 104 and 107; Brescovit et al. 2019: figs. 26–30). Females can be recognised by the boat-shaped structure on the postepigastric scutum (Fig. 6G).

#### Distribution

Pantropical; newly recorded from China.

## Supplementary Material

XML Treatment for Ischnothyreus daheling

XML Treatment for Ischnothyreus longyang

XML Treatment for Ischnothyreus velox
